# Rigidifying a *De Novo* Enzyme Increases Activity and Induces a Negative Activation Heat Capacity

**DOI:** 10.1021/acscatal.1c01776

**Published:** 2021-09-01

**Authors:** Sarah A. Hindson, H. Adrian Bunzel, Bettina Frank, Dimitri A. Svistunenko, Christopher Williams, Marc W. van der Kamp, Adrian J. Mulholland, Christopher R. Pudney, J. L. Ross Anderson

**Affiliations:** † Department of Biology and Biochemistry, Centre for Sustainable Chemical Technology, University of Bath, Bath BA2 7AY, U.K.; ‡ School of Biochemistry, University of Bristol, Bristol BS8 1TD, U.K.; § Centre for Computational Chemistry, University of Bristol, Bristol BS8 1TS, U.K.; ∥ Bristol Centre for Functional Nanomaterials, School of Physics, University of Bristol, Bristol BS8 1TL, U.K.; ⊥ School of Life Sciences, University of Essex, Colchester CO4 3SQ, U.K.

**Keywords:** enzyme catalysis, protein dynamics, peroxidase, C45, MMRT, REES, activation heat capacity

## Abstract

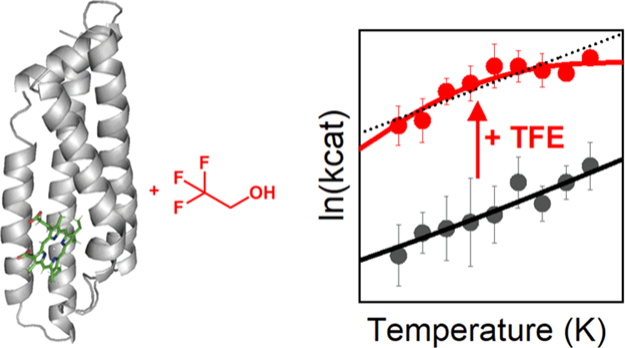

Conformational sampling profoundly impacts the overall activity and temperature dependence of enzymes. Peroxidases have emerged as versatile platforms for high-value biocatalysis owing to their broad palette of potential biotransformations. Here, we explore the role of conformational sampling in mediating activity in the *de novo* peroxidase C45. We demonstrate that 2,2,2-triflouoroethanol (TFE) affects the equilibrium of enzyme conformational states, tending toward a more globally rigid structure. This is correlated with increases in both stability and activity. Notably, these effects are concomitant with the emergence of curvature in the temperature-activity profile, trading off activity gains at ambient temperature with losses at high temperatures. We apply macromolecular rate theory (MMRT) to understand enzyme temperature dependence data. These data point to an increase in protein rigidity associated with a difference in the distribution of protein dynamics between the ground and transition states. We compare the thermodynamics of the *de novo* enzyme activity to those of a natural peroxidase, horseradish peroxidase. We find that the native enzyme resembles the rigidified *de novo* enzyme in terms of the thermodynamics of enzyme catalysis and the putative distribution of protein dynamics between the ground and transition states. The addition of TFE apparently causes C45 to behave more like the natural enzyme. Our data suggest robust, generic strategies for improving biocatalytic activity by manipulating protein rigidity; for functional *de novo* protein catalysts in particular, this can provide more enzyme-like catalysts without further rational engineering, computational redesign, or directed evolution.

Biocatalysis is central to the development of a sustainable chemical industry.^1^ Many natural enzymes are extremely proficient, highly specific, and can provide access to novel and efficient synthetic routes. Nonetheless, their biocatalytic applications are limited by the exquisite selectivity that enzymes display for their natural transformations. Creation of novel enzymes through *de novo* design or directed evolution provides a means to fill such gaps in the biocatalytic toolbox.^2−5^ These engineering efforts often have significant effects on the scaffold dynamics,^5^ and a complete understanding of the molecular and dynamic changes wrought by engineering—particularly the effects on the thermodynamic drivers of catalysis—is required to rationally create better enzymes in the future.

Heme peroxidases are versatile biocatalysts that exploit their heme cofactor to form highly oxidative oxyferryl intermediates (Figure 1B).^6^ We have recently created the *de novo* heme-peroxidase C45 (Figure 1A), a rationally designed four-helical bundle protein with high peroxidase activity.^7^ Notably, C45 activity approaches that of natural benchmarks such as horseradish peroxidase (HRP) but is limited by the lack of a catalytic proton-shuttling residue, which likely slows down both the initial H_2_O_2_ deprotonation and the subsequent cleavage of the peroxide O–O bond; this results in an elevated *K*_M_ for H_2_O_2_.^7^ The relative simplicity of these *de novo* designed helical bundles allows facile tailoring for specific purposes, e.g., by introducing site-specific functional moieties.^8−11^ Furthermore, they are often highly flexible, facilitating access to a broad equilibrium of conformational states for engineering.^12^ In contrast to many other heme-dependent enzymes, C45 catalyzes a broad range of transformations such as dehalogenations and carbene-transfer reactions.^13^

**Figure 1:**
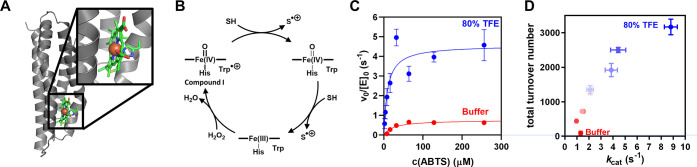
TFE boosts the activity of *de novo* heme-peroxidase C45. (A) The heme cofactor of C45 (green sticks, iron: brown sphere) gives rise to catalytic peroxidase activity (B; proposed catalytic cycle). (C) Addition of 80% TFE (blue) boosts oxidation of ABTS by C45 compared to buffer (red). (D) TFE additionally increases the total turnover number (TTN) of C45 (red to blue: 0–80% TFE). Conditions: 25–200 nM C45, 100 μM H_2_O_2_, 100 mM KCl, 20 mM CHES, pH 8.6, 25 °C.

Understanding the relationship between protein dynamics and enzyme turnover holds great promise for the ability to engineer enzyme activity.^5,14−17^ Moreover, there are important fundamental questions regarding the relationship between the global protein flexibility and the conformational dynamics that are relevant to enzyme turnover. Recent efforts to understand the relationship between protein flexibility (the equilibrium of conformational states; dynamics) and enzyme turnover have employed macromolecular rate theory (MMRT)^12,18^ to infer differences in the distribution of vibrational modes between the ground and transition state. Evidence from a range of experimental and computational studies points to the involvement of networks of protein dynamics that extend throughout the protein and are not just localized to the immediate active site.^19−21^ MMRT quantifies the difference in the distribution of vibrational modes between the ground and transition state ensemble as the heat capacity of catalysis (Δ*C*_P_^‡^) given by
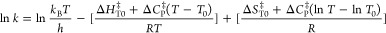
1where Δ*H*_T0_^‡^ and Δ*S*_T0_^‡^ are the activation enthalpy and entropy at an arbitrary reference temperature *T*_0_. Where such curvature is present, values of Δ*C*_P_^‡^ are typically negative for natural enzymes, manifesting as concave curvature in plots of ln *k* versus *T*.

Here, we provide evidence that global rigidification of C45 increases enzyme activity and also induces a difference in rigidity (dynamics) between the ground and transition state ensembles. By comparison to a natural enzyme, our data point to a complex interplay between enzyme activity, stability, and protein dynamics. We discuss the implications for protein engineering principles that might leverage this emerging relationship.

## Results and Discussion

### Trifluoroethanol Increases Peroxidase Activity

When studying the effects of various organic co-solvents on C45 peroxidase activity, we observed that addition of 2,2,2-trifluoroethanol (TFE) increased enzyme activity (Figure 1C). While other co-solvents had a deleterious effect on turnover (Figure S1), addition of 80% TFE resulted in a 6-fold increase in peroxidase activity for ABTS (2′-azino-bis(3-ethylbenzothiazoline-6-sulfonic acid)), as reflected in the increased *k*_cat_ under limiting peroxide concentration from 1.3 ± 0.2 to 7.9 ± 0.9 s^–1^ (Figure 1C). Notably, TFE not only increases the rate of C45 turnover but also increases its total turnover number (TTN) by up to ∼35-fold in 80% TFE (Figure 1D).

We have monitored the stability of the catalytic competency of C45 from steady-state progress curves, as shown in Figure S2. At low temperatures, our progress curves are linear for >10 min; however, at elevated temperatures, there is apparent curvature. In the absence of trivial effects such as substrate depletion, this finding is typically indicative of enzyme inactivation/unfolding. From Figure S2, the curvature is rather more pronounced in the absence of TFE on the same timescale, suggesting that TFE stabilizes the catalytically competent conformational state of C45. Our data therefore suggest that the reason for the higher TTN in the presence of TFE arises not just because of a faster rate of turnover and more stable intermediate but also at least in part a more stable enzyme. See below for a further discussion on C45 stability with TFE.

Peroxidases form highly reactive oxyferryl intermediates that may oxidize and damage the protein and/or the heme cofactor,^6^ and the apparent increase in TTN suggests that in 80% TFE, C45 favors productive turnover over such detrimental off-pathway reactions. There is a range of potential mechanisms through which we can envisage TFE acting, including stabilization of Compound I and/or prevention of heme degradation via protein rigidification, which inhibits the reaction with neighboring side chains; ABTS binding more readily to the protein surface in TFE, similarly preventing a Compound I degradation.

**Figure 2:**
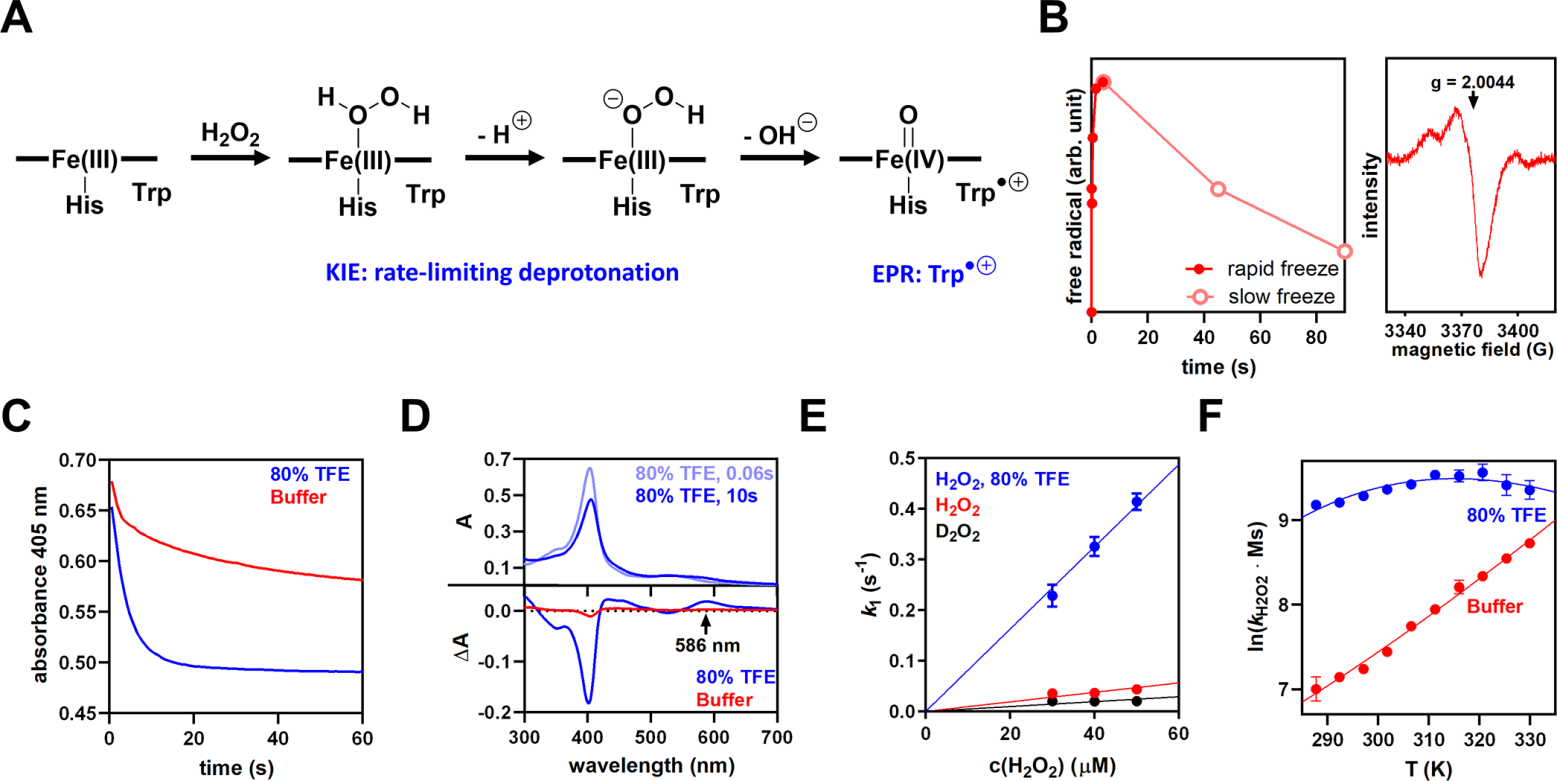
Accelerated formation and increased stability of Compound I in C45 in the presence of TFE. (A) Proposed catalytic mechanism for formation of Compound I in C45. (B) Formation and degradation of a radical species can be observed by EPR spectroscopy upon addition of H_2_O_2_ to C45. Inset: EPR spectrum of the Trp radical intermediate (also see Figure S3B). Conditions: 67.5 μM C45, 13 μM H_2_O_2_, 25 °C. (C) TFE accelerates Compound I formation and heme degradation, as indicated by biphasic pre-steady-state kinetics. (D) Top: UV/Vis spectra in 80% TFE at 0.06 s (light blue) and 60 s (dark blue). Bottom: Corresponding difference spectra for 80% TFE (blue) and buffer (red). Compound I has a Q band at 586 nm that becomes most pronounced in 80% TFE at high temperatures. Conditions C + D: 5 μM C45, 50 μM H_2_O_2_, 60 °C. (E) Rates of intermediate formation determined in 80% TFE (blue), buffer (red), and 80% D_2_O. Conditions: 1 μM C45, 5 °C. (F) Temperature dependence of Compound I formation in 80% TFE (blue) and buffer (red). Conditions: 2.5 μM C45, 25 μM H_2_O_2_. All experiments were measured in 100 mM KCl, 20 mM CHES, pH 8.6, with either 80% D_2_O or 80% TFE.

To probe the mechanistic underpinning for the enhanced activity, we determined the kinetics of Compound I formation using pre-steady-state kinetics (Figure 2). H_2_O_2_ turnover in the presence of C45 proceeded with multiphasic kinetics, with the first phase relating to formation of intermediate and subsequent phases to its decay and heme degradation as indicated by an overall UV/Vis-absorbance bleach. EPR spectroscopy allowed to electronically characterize that intermediate after freeze-quenching following addition of H_2_O_2_ (Figures 2B and S3B). An anisotropic EPR signal at *g* = 2.0044 was observed; its line shape is consistent with that observed in a dye decolorizing peroxidase and attributed to a tryptophan radical.^22^

The rate of Compound I formation (*k*_H2O2_) can be quantified from the observed rate in the peroxide concentration dependence (Figure 2E). Notably, performing the reaction in 80% D_2_O decreased *k*_H2O2_ from 940 ± 30 to 490 ± 20 M^–1^ s^–1^. The resulting kinetic isotope effect of 1.9 implies that the rate-limiting step in Compound I formation is the abstraction of a proton that exchanges with the solvent, which probably represents the initial deprotonation of hydrogen peroxide at the distal heme face. These observations are in good agreement with the high *K*_M_ for H_2_O_2_ and may be related to the lack of distal histidine residues in C45, which are commonly employed in natural peroxidases to perform this deprotonation and to facilitate proton shuttling.

From Figure 2E,F, the addition of TFE gave rise to an increase in *k*_H2O2_ and a significant reduction in the rate of decay of Compound I in the absence of ABTS. TFE increases *k*_H2O2_ 9-fold compared to the reaction in buffer to 8130 ± 80 M^–1^ s^–1^, mirroring the increased ABTS oxidation activity. Notably, addition of TFE also decreased heme degradation by up to 2-fold at 5 °C. The apparent stabilization of the reactive Compound I intermediate allows C45 to maintain activity over a prolonged time, which directly relates to the increase in the total turnover number observed for ABTS oxidation. Intriguingly, we observed the highest accumulation of Compound I at elevated temperatures (60 °C) in TFE. Under these conditions, a new Q band (586 nm) is clearly observable (Figures 2D and S3A). Our kinetic data therefore indicate that the rate of Compound I formation, as well as its stability, is improved in the presence of TFE. The unexpectedly high stability of Compound I at high temperatures may point to changes in the temperature dependence arising from thermodynamic perturbation of the system. Figure 2F shows the temperature dependence of Compound I formation in the presence and absence of TFE. From Figure 2F, the presence of TFE gives a much “shallower” temperature dependence, which curves. We discuss these data in detail below.

### Structural and Dynamical Effects of TFE

TFE is known to stabilize α-helical folds by strengthening intramolecular hydrogen bonds and hydrophobic interactions^23,24^ and appears to drive the adoption of a more active catalytic geometry in C45 (Figure 1). We therefore hypothesized that, in the absence of TFE-induced conformational change, the rigidification of C45 might be the driver of changes in Compound I formation and stability that give rise to the enhanced *k*_cat_ values. We employed a range of spectroscopic methods to probe the putative restriction of conformational dynamics of C45 upon addition of TFE.

**Figure 3:**
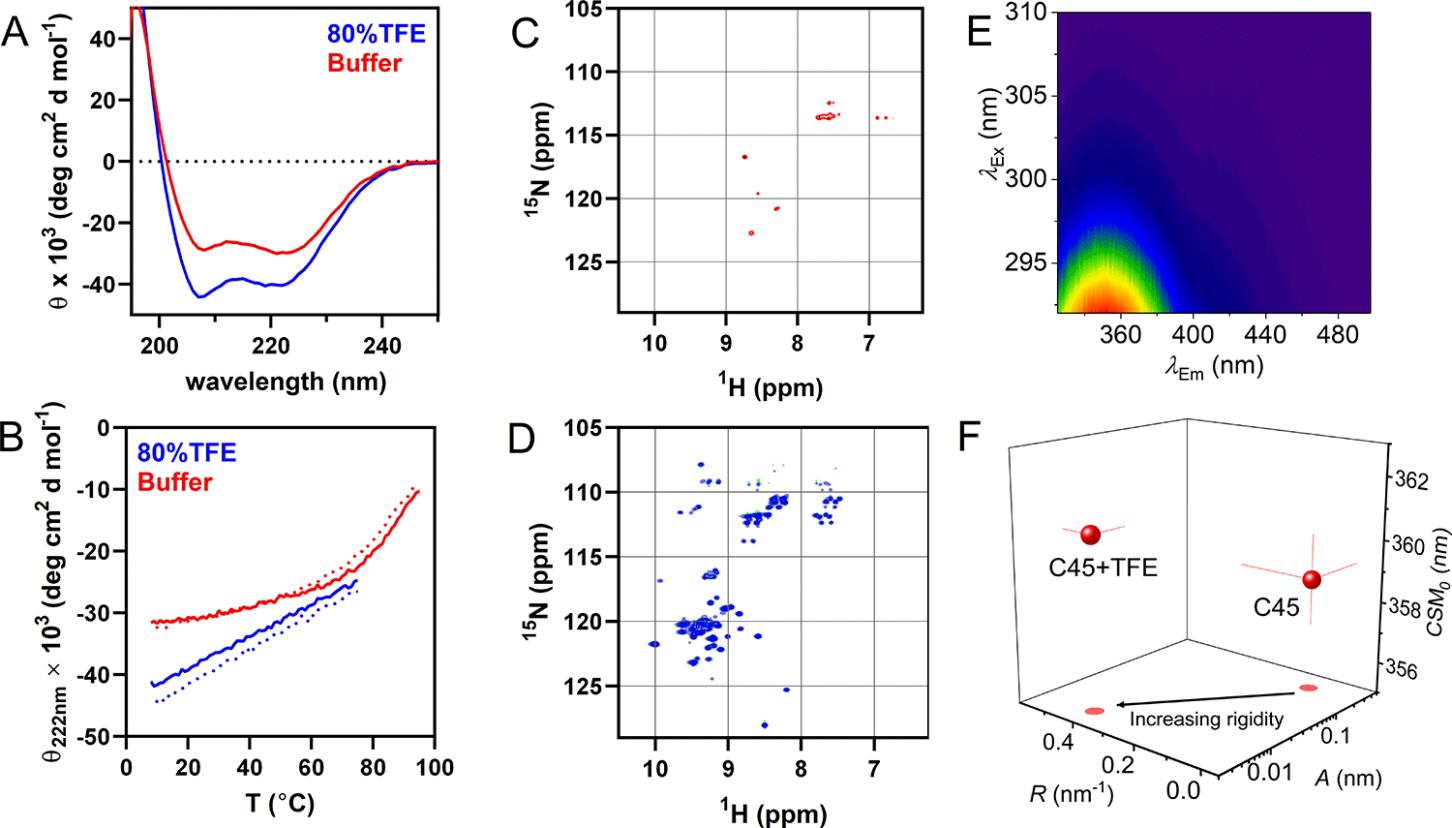
C45 rigidification in TFE. (A) CD spectra and (B) melting curves of C45 in buffer (red) and 80% TFE (blue). The dotted lines show the reversible refolding. Conditions: 5–10 μM C45, 100 mM KCl, 20 mM CHES, pH 8.6. (C, D) ^1^H–^15^N TROSY-HSQC NMR spectra of C45 in buffer (panel C; red) and in 80% TFE (panel D; blue). Conditions: 250 μM C45, 100 mM KCl, 20 mM CHES, pH 8.6, 10% D_2_O. (E) Example contour plot showing the change in the structure of the emission spectra with increasing λ_Ex_. (F) QUBES data for C45 in buffer and in 50% TFE resulting from the fits shown in Figure S4. The solid black arrow indicates a decrease in the ratio *A*/*R*, interpreted as an increase in protein rigidity. Conditions: 4 μM C45 in 50 mM HEPES buffer, pH 6.5, 15 °C.

Far-UV CD spectra (Figure 3A) suggest an enhanced helical structure content in the presence of TFE, with a more negative ellipticity value at 222 nm (θ_222__nm_). Thermal melts monitoring θ_222__nm_ in the absence of TFE (Figure 3B; red line) show a progressive increase in θ_222__nm_, with an upward curvature that is indicative of relatively uncooperative unfolding of the helical structure. In the presence of TFE (Figure 3B; blue line), we only observe a linear increase in θ_222__nm_ with almost no curvature. That is, we do not observe the beginning of an unfolding transition as observed in the absence of TFE. These data track with our previous work in showing that the secondary structure of C45 is extremely thermally stable^7^ but that this stability is enhanced in the presence of TFE. Moreover, these data are consistent with our kinetic progress curves (Figure S2) in suggesting that TFE stabilizes C45 with respect to denaturation. Addition of other co-solvents such as methanol, ethanol, and acetonitrile significantly decreased activity (Figure S1). Indeed, for both MeOH and EtOH, the CD spectra and resulting temperature dependence of the CD signal are effectively identical to C45 in the presence of TFE. These data therefore suggest that TFE serendipitously traps C45 in a more active conformational ensemble, rather than the enhanced rate of turnover and TTN being associated with a different structural form associated with increased stability of helices.

NMR spectroscopy allowed us to monitor the putative stabilizing effect of TFE at the residue level. The two-dimensional ^1^H–^15^N TROSY-HSQC spectrum of C45 recorded in the absence of TFE (Figures 3C and S4) demonstrates relatively poor signal dispersion and resolution, suggesting a high degree of conformational flexibility, consistent with our previously reported C45 spectra and common to *de novo* designed helical bundles.^7,25,26^ In contrast, the addition of TFE to C45 results in a ^1^H–^15^N TROSY-HSQC spectrum (Figure 3D) with a notably increased number of sharper peaks. These data indicate that not only does C45 become more stable but is also less flexible/more compact. Rigidification slows down T2 relaxation, which increases the intensity and sharpens peaks. The increased peak dispersion signals that C45 exists in a more defined conformational ensemble in TFE.

Far-UV CD cannot directly capture changes in protein flexibility and the protein concentrations used for NMR are high, compared to our kinetic studies, which potentially can affect molecular flexibility via macromolecular crowding.^27^ We therefore turned to our recent work using the red-edge excitation-shift (REES) phenomenon to further explore the change in C45 flexibility induced by TFE. Briefly, the REES phenomenon is observed as the inhomogeneous broadening of emission spectra with a decrease in excitation energy. This broadening reflects the presence of discrete solvent–solute interaction energies that are increasingly photoselected for by the change in excitation energy.^28^ We have demonstrated that this effect is capable of tracking changes in protein conformational sampling by monitoring intrinsic tryptophan emission, via a simple numerical model of the underlying REES effect.^29−31^ Indeed, we have demonstrated that this approach can identify differences in conformational sampling even between essentially identical crystal structures.^30^ We term our quantification and interpretation of the REES effect, Quantitative Understanding of Bimolecular Edge Shift (QUBES). We track changes in the broadening of Trp emission spectra as the change in the center of spectral mass (CSM; see Materials and Methods)
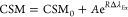
2where the amplitude and curvature of the exponential are described by *A* and *R* values, respectively. CSM_0_ is the CSM value independent of λ_Ex_, the excitation wavelength, and Δλ_Ex_ is the change in excitation wavelength from 292 nm. CSM_0_ is then analogous to the use of Trp emission spectra to track changes in solvent exposure and infer tertiary structural changes, i.e., as an increase in emission wavelength on unfolding (solvent exposure).^32^ That is, an increase in CSM_0_ would imply unfolding of the protein, increased solvent exposure of Trp. We have previously found that, for an invariant CSM_0_ value, an increased *A*/*R* value reflects a broader population of conformational states.^30,31^

Figure 3E shows the example raw REES data as the matrix of emission spectra versus changes in excitation energy. Plots of CSM versus excitation energy are shown in Figure S5, with the resulting parameters from fits to eq 2 shown in Figure 3F. We note our tryptophan REES data are free from convolution with any iron-free heme, as described in the Materials and Methods section and shown Figure S5. From Figure 3F, the CSM_0_ is the same within error and the *A*/*R* value in the presence of TFE is significantly smaller compared the absence of TFE. These data therefore suggest that the presence of TFE narrows the distribution of conformational states of C45. Potentially, the narrower distribution of conformational states is the cause of the C45 rigidification in TFE. Moreover, our data do not suggest a measurable change in the overall structure, since the extracted CSM_0_ values are essentially identical in the presence and absence of TFE (Figure 3F).

### Thermodynamic Effects of Altered C45 Flexibility

**Figure 4:**
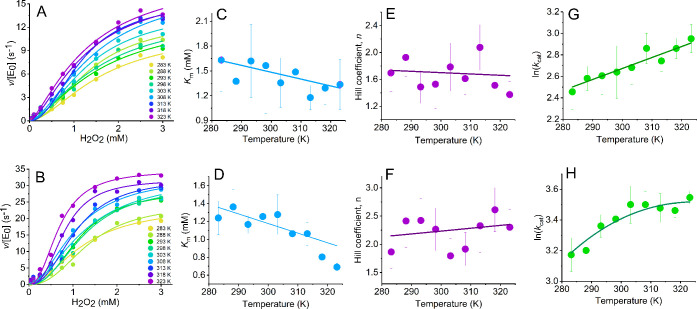
Temperature dependence of C45 turnover in the presence (bottom) and absence (top) of TFE. (A, B) Concentration dependence of H_2_O_2_ versus rate of C45 turnover with increasing temperature (10–50 °C) in the absence (panel A) and presence of TFE (panel B). Solid lines are the fits of the data to eq 3. (A) in buffer and (B) with 50% TFE (v/v). (B–H) Temperature dependence of parameters extracted from steady-state data (panels A and B) for *n* (panels C and D), *K*_M_ (panels E and F), and *k*_cat_ (panels G and H). Solid lines in panels (C–F) are fits to a simple linear function and are to aid the eye only. Solid lines in panel (G, H) are the fit to eq 1. The resulting parameters are given in Table 1. Conditions: 0.154 μM C45 and 73 mM ABTS in 50 mM HEPES buffer, pH 6.5.

To understand how the apparent protein rigidification affects the thermodynamic drivers of catalysis, we analyzed the temperature dependence of C45 steady-state turnover (Figure 4 and Table 1). From our previous studies, we do not expect strong binding of ABTS to C45.^7^ We have therefore conducted our steady-state kinetic analysis at the highest ABTS concentrations practically achievable. From Figure 4A,B, at all temperatures studied and both in the presence and absence of TFE, the steady-state kinetic data show a sigmoidal relationship, implying non-Michaelis–Menten-type kinetics. These data contrast our work with C45 conducted at lower ABTS/H_2_O_2_ concentrations and/or a different buffer system, suggesting that the elevated substrate concentrations and/or the change in the buffer system have exposed a kinetic relationship that was not otherwise readily detectable. We note that to compare the kinetics of C45 to a natural enzyme (see below), we use a different buffer system with a much lower pH compared to the previous reports^7^ and so we do not expect the absolute magnitude of the extracted kinetics to be identical.

**Table 1:** Kinetic and Thermodynamic Parameters Extracted from Steady-State Kinetics for Both C45 and HRP

	C45	C45 + 50% TFE	HRP
*k*_cat H2O2_ (s^–1^)	13.5 ± 2.4	28.8 ± 2.1	638.0 ± 45
*K*_m H2O2_ (mM)	1.6 ± 0.40	1.2 ± 0.10	0.05 ± 0.01
*n*	1.6 ± 0.2	2.1 ± 0.3	
*T*_opt_ (K)		325.5	321.2
Δ*C*_P_^‡^ (kJ mol^–1^ K^–1^)	0.04 ± 0.19	–0.28 ± 0.19	–1.65 ± 0.75
Δ*H*_T0_^‡^ (kJ mol^–1^)^a^	5.4 ± 0.95	3.0 ± 0.90	24.1 ± 4.70
Δ*S*_T0_^‡^ (kJ mol^–1^ K^–1^)^a^	1.1 ± 0.01	1.1 ± 0.01	1.2 ± 0.02

a *T*_0_ = 303 K.

It is common^33^ to fit such apparently sigmoidal steady-state data to the Hill equation
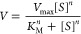
3where the Hill coefficient, *n*, captures the deviation from hyperbolic Michaelis–Menten curves. The Hill coefficient is often attributed to allosteric binding effects.^34,35^ The results of fitting our data to eq 3 are given in Table 1. From Table 1, as with our findings above, the extracted *k*_cat_ is larger in the presence of TFE (Table 1). The magnitude of *n*, both in the absence and presence of TFE, signals positive cooperativity; *n* = 1.6 ± 0.2 and 2.1 ± 0.3 at 293 K, respectively, noting that these values are within error of each other. From Figure 4C,D, we find the magnitude of *n* is essentially invariant with temperature within the error of the measurement, in both the presence and absence of TFE. The extracted *K*_M_ values are again similar in the presence and absence of TFE (Figure 4E,F; Table 1); *K*_M_ = 1.6 ± 0.4 and 1.2 ± 0.1 mM, respectively. The *K*_M_ shows some decrease with the increasing temperature, but this is the same within error in both the presence and absence of TFE, being −0.01 ± 0.003 and −0.008 ± 0.003 mM K^–1^, respectively. In summary, the magnitude and observed temperature dependence of both *n* and *K*_M_ are the same within the error of the measurement in the presence and absence of TFE. That TFE has no measurable effect of either the magnitude or temperature dependence of *n* and *K*_M_ is powerful evidence that TFE does not alter the rate-limiting step since both of these parameters are acutely sensitive to such a change.

We acknowledge that diagnosing true allosteric cooperativity is challenging, not at least for a small artificial enzyme system where one of the substrates does not have an obvious formal binding site, as we describe above. Without a structural underpinning for the effect, we cannot consider the detailed putative allosteric mechanism. Instead, accurately tracking the steady-state kinetics of C45 allows us to extract robust kinetic data, from which we are able to determine key thermodynamic parameters from temperature dependence studies.

The temperature dependence of the extracted *k*_cat_ values are shown in Figure 4G,H in the presence and absence of TFE (50% v/v), respectively. The extracted parameters resulting from fits to eq 1 are given in Table 1. From Figure 4G,H and Table 1, we find there is evident curvature in the temperature dependence plots in the presence of TFE only, manifesting as a shift from a ∼zero Δ*C*_P_^‡^ (0.04 ± 0.19 kJ mol^–1^ K^–1^) to a measurably negative Δ*C*_P_^‡^ in the presence of TFE (−0.28 ± 0.19 kJ mol^–1^ K^–1^). The extracted Δ*C*_P_^‡^ for ABTS formation is reminiscent of our observation for the temperature dependence of Compound I formation (Figure 2F), which gives Δ*C*_P_^‡^ = −0.58 ± 0.22 and 0.19 ± 0.11 kJ mol^–1^ K^–1^ in the presence and absence of TFE, respectively. That is, we find a similar trend in TFE inducing a negative Δ*C*_P_^‡^ from both steady-state kinetics and pre-steady-state kinetics. These data would strongly argue that the observed curvatures in the temperature dependence of ln* k* in the presence of TFE are not due to a shift in the rate-limiting step of turnover and moreover that the negative Δ*C*_P_^‡^ in the presence of TFE may be related to Compound I formation.

Given we have demonstrated that C45 is more stable with TFE present (above), the apparent negative Δ*C*_P_^‡^ value in the presence of TFE would not appear to be due to local unfolding. Our studies with alternative co-solvents (Figure S1) suggest that the increased helicity we observe is not associated with the observed changes in turnover and so rule out an alternate structural form giving rise to curvature. We note that we cannot explicitly rule out contributions from more complex causes, e.g., quantum mechanical tunneling contributions. However, this seems unlikely, given the mechanism appears to be unaffected by the presence of TFE inferred from our observation of Compound I formation (Figure 2). Moreover, our stopped-flow and absorption data studies (discussed above) indicate that the chemical mechanism is essentially invariant on addition with TFE and that that there is no change in the rate-limiting step. In the absence of such confounding factors, a negative Δ*C*_P_^‡^ indicates a difference in the distribution of vibrational modes between the ground and transition states: In a simple sense, a difference in the selective rigidification between the ground and transition state ensemble. Given our data above (Figure 3) show that the effect of TFE is to “rigidify” C45, it is a logical conclusion that this rigidification is causal in altering the distribution of vibrational modes that gives rise to an observable negative Δ*C*_P_^‡^. Based on the previous work, we anticipate this could involve larger-scale motions throughout the protein as illustrated previously in other systems,^19,36^ though we do not rule out more localized effects.

The presence of TFE induces a negative Δ*C*_P_^‡^, which manifests as curvature in the temperature dependence of *k*_cat_. A corollary of this is the presence of a temperature optimum of the reaction (*T*_opt_) in the presence of TFE (*T*_opt_ = 325.5 K). Given that there is no measurable curvature in the temperature dependence data in the absence of TFE, we cannot assign a *T*_opt_. Our data therefore indicate that while TFE increases the thermal stability of C45, it also induces a *T*_opt_ at a temperature much lower than unfolding (by comparisons to our CD data; Figure 3). Rigidifying the enzyme, induced by TFE, appears to decouple the stability of C45 and the temperature optimum of enzyme turnover.

### Comparison to the Kinetics and Thermodynamics of a Natural Peroxidase

We wish to explore the effect of a globally different relationship of protein conformational dynamics to enzyme turnover, beyond what is accessible with our TFE studies. To that end, we compare C45 to a natural enzyme that catalyzes a similar chemical reaction, albeit with a more typical (larger) protein structure. Horseradish peroxidase (HRP) is an excellent model system for this purpose. HRP is ∼44 kDa with a largely α-helical structure, with peroxidase activity mediated by a *b*-type heme cofactor.^37^

First, we assessed the role, if any, of protein conformational dynamics on HRP turnover. While HRP can potentially be stable to TFE,^38^ we find that in our buffer system this is not the case and the extracted rate constant decreases with increasing TFE (Figure S6), presumably due to HRP unfolding. Instead of using TFE as with C45, we use combined temperature and pressure studies to alter the equilibrium of conformational states in an effort to provide more detailed insights into the conformational state equilibria affecting turnover.

**Figure 5:**
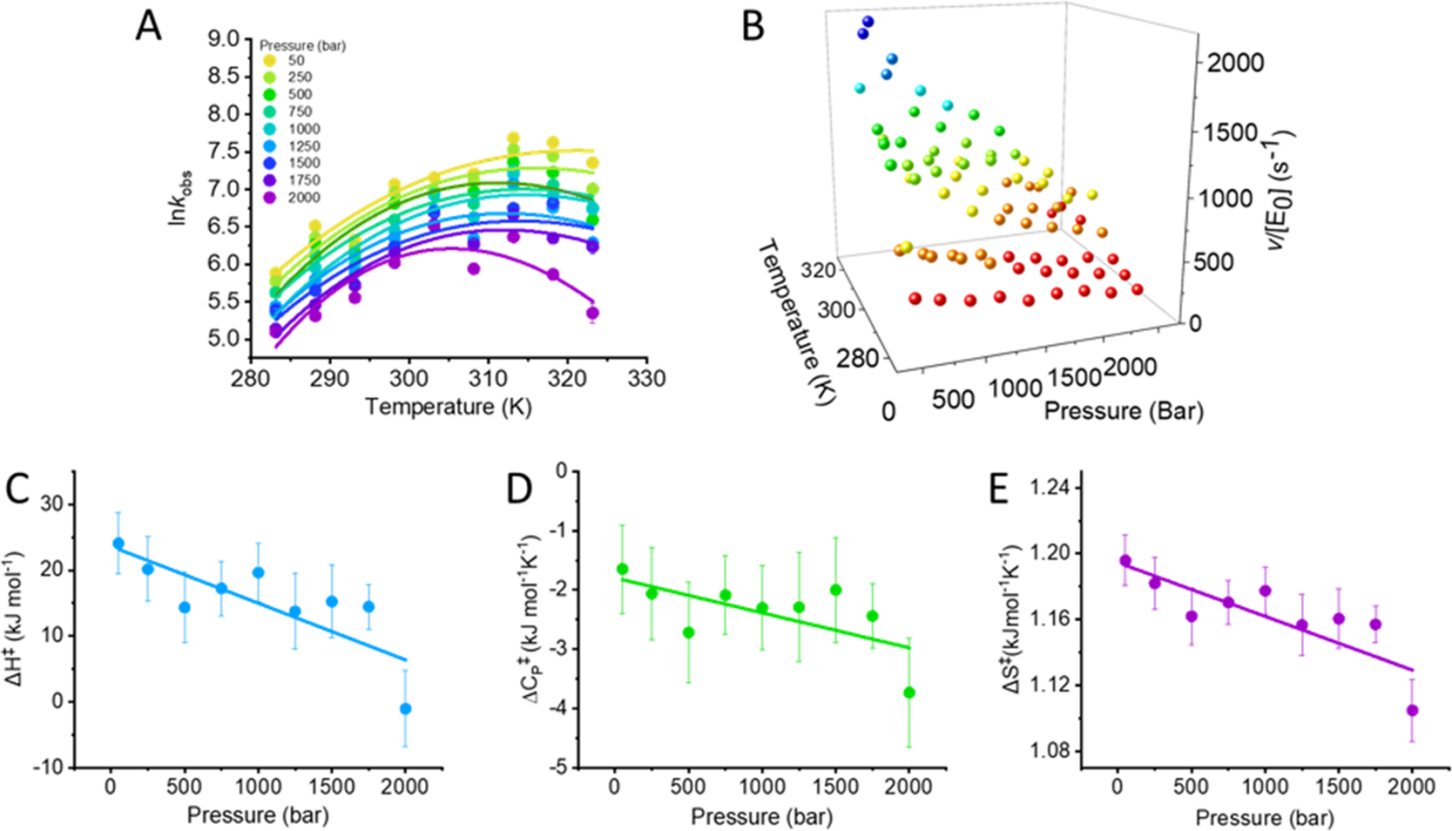
Combined pressure and temperature dependence of HRP turnover (panels A and B). The solid lines in panel (A) are the fits to MMRT eq 1. (C–E) Pressure dependence of resulting parameters from fits in panel (A). The solid lines are to aid the eye only and illustrate the prevailing trend in the data with the resulting parameters plotted in panels (C–E). The resulting parameters are given in Table 1. Conditions: 0.2 nM HRP, 1.8 mM H_2_O_2_, and 73 mM ABTS in 50 mM HEPES buffer, pH 6.5.

Nondenaturing hydrostatic pressure is an established method for demonstrating the sensitivity of enzyme turnover to changes in the equilibrium of protein conformational states.^39−42^ As a broad framework, one expects a significant pressure dependence on enzyme activity, in cases where the turnover is affected by the protein’s conformational dynamics.^43−45^ We have previously demonstrated a correlation of a pressure-dependent Δ*C*_P_^‡^ with differences in global protein flexibility.^30^Figure 5A,B shows the combined pressure–temperature dependence of *k*_cat_ for HRP and the resulting pressure dependence of the values extracted by fitting to eq 1 and given in Table 1. Example steady-state Michaelis–Menten data are shown in Figure S7. We note that our progress curves are linear at elevated pressures (Figure S8), and our data are fully reversible with pressure, showing that our data are not convolved with unfolding at the pressures we use.

From Figure 5A,B, the *k*_cat_ of HRP varies significantly with both pressure and temperature. That is, we observe a pressure-dependent change in *k*_cat_ at all temperatures studied, suggesting that altering the distribution of conformational states impacts on HRP turnover and in turn implying a role for HRP’s conformational dynamics in enzyme turnover. We note that the substrates in the absence of the enzyme show essentially no chemical turnover on the timescales of our assays, and so the pressure dependence we observe is due to the enzyme.

Our pressure/temperature matrix (Figure 5A,B) allows us to further explore whether the thermodynamics associated with enzyme turnover are pressure-dependent and to more specifically infer the role of protein conformational dynamics. Figure 5C,D shows the pressure dependence of the extract values from eq 1 at each pressure studied. From these data, it is clear that all thermodynamic parameters are pressure-dependent, showing an approximately linear decrease with respect to pressure. It is interesting to note that the decrease in Δ*C*_P_^‡^ is also correlated with a general decrease in *T*_opt_ (Figure 5A), consistent with the predictions from MMRT. These data therefore suggest that altering the equilibrium of protein conformational states in HRP is sufficient to alter the thermodynamics of enzyme turnover, analogous to the effect of TFE on C45.

From Table 1, the kinetics and thermodynamics of C45 turnover in the presence of TFE approach those of HRP; with an elevated *k*_cat_, a measurably negative Δ*C*_P_^‡^ and a similar *T*_opt_. It is therefore tempting to speculate that the rigidification of C45 has given rise to a more “natural”-like enzyme at least in terms of the thermodynamics of enzyme turnover. This suggestion is satisfying because one anticipates that enzyme active sites are significantly organized by the bulk protein.

## Conclusions

*De novo* enzymes have enormous potential to be platform biocatalysts, with significant scope for engineering and tuning activity. It is common for enzyme engineering efforts with “natural” enzymes to attempt to rigidify the overall structure/active site, primarily for enhanced thermal stability, but also to precisely engineer specific active site geometries. Here, we use a model peroxidase with excellent catalytic activity to explore the effect of altering the rigidity of an artificial enzyme on protein dynamics, stability, and catalytic activity. By using a simple shift in the solvent system, we are able to tune the rigidity of C45, not only increasing thermal stability but also enhancing the rate of turnover. The combined effect of increased stability and activity leads to an enzyme with a significantly increased total turnover number compared to the parent system. Potentially, rigidification of C45 drives the adoption of a more active state, e.g., by stabilization of Compound I, as we discuss above. That a simple solvent change is able to increase the TTN of the *de novo* enzyme by over 30-fold points to a potentially simple inexpensive route to tune such enzymes. We note that the link between protein flexibility/rigidity and activity will be system dependent but smaller, less complex systems like C45 provide a window into this relationship. Indeed, other artificial heme-enzyme systems have been reported to have increased activity in the presence of TFE.^46,47^ However, we do not suggest that co-solvent variation is a generalizable tool to affect the flexibility/rigidity of all enzymes but illustrates the principle that tuning flexibility can be used to tune activity.

Our thermodynamic studies point to a most intriguing finding, namely that increasing the rigidity of C45 decouples thermal stability from the temperature optimum of reaction. That is, for C45 we observe global rigidification of the protein at large but simultaneously a difference in the distribution of vibrational modes between the ground and transition states, giving rise to a negative Δ*C*_P_^‡^ and a measurable *T*_opt_, well below a measurable unfolding transition. C45 is therefore a key case study in illustrating that the anticipated relationship between stability, activity, and response to temperature does not always track in the expected “textbook” way. Moreover, it is notable that these findings are with a small, *de novo* enzyme versus a large natural enzyme. This study adds to the growing evidence that a negative activation heat capacity is indicative of “enzyme-like” behavior and can correlate with catalytic activity.^48^

Comparisons of the thermodynamics of reaction between C45 and a natural enzyme (HRP) show that for both proteins, perturbing the equilibrium of conformational states can alter the thermodynamics of turnover and particularly the magnitude of Δ*C*_P_^‡^. This finding is all of the more interesting since the rigidification appears to tune the thermodynamics of C45 to be more similar to a larger “natural” enzyme, which catalyzes a similar chemical reaction. Our data then provoke important questions around the “optimal” flexibility of an enzyme and the role of the protein scaffold at sites distal to the immediate active site volume.^13^

## Materials and Methods

### Protein Production and Purification

C45 was produced and purified, as previously described.^7^ Briefly, C45 was expressed in T7 express *Escherichia coli* BL21 (DE3) co-transformed with vector pEC86 encoding for the c-type cytochrome maturation system Ccm. Protein expression was induced with the addition of isopropyl β-d-1-thiogalactopyranoside (IPTG). A cell pellet was collected and lysed, then purified using nickel affinity column chromatography. The 6-histidine tag was cleaved using tobacco etch virus protease (100 μg L^–1^ expression culture) under anaerobic conditions in the presence of a reducing agent (1 mM Tris(2-carboxyethyl)phosphine). The cleaved protein was further purified using nickel affinity column chromatography and size exclusion chromatography, flash-frozen using liquid nitrogen, and stored at −70 °C. HRP was purchased from Sigma-Aldrich.

### CD Spectroscopy

CD experiments were carried out on a Jasco J-810 spectrophotometer, using a sealed quartz cuvette. A baseline of the buffer solution including co-solvent was obtained and subtracted from protein measurements. Scans were taken at a rate of 100 nm per minute and a temperature of 25 °C. Thermal denaturation studies were conducted with a ramp of 1 °C min^–1^, starting at 8 °C.

### REES Spectroscopy

All fluorescence measurements were performed using a Perkin Elmer LS50B Luminescence Spectrometer (Perkin Elmer, Waltham, MA) connected to a circulating water bath for temperature regulation (1 °C). Samples were thermally equilibrated by incubation for 5 min at the given conditions prior to recording measurements. For all samples, the corresponding buffer control was subtracted from the spectra for each experimental condition and this also removed the Raman water peak.

The fluorescence emission spectra are typical of Trp fluorescence data, with the exception that an additional minor spectral feature at ∼440 nm that can be attributed to iron-free heme. The quantification of the REES data relies on accurate extraction changes to the emission spectra and so the additional band would convolve the measurement. We have therefore numerically modeled each of the spectra using a sum of two skewed Gaussians
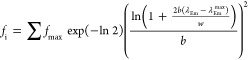
4where *f*_i_ is the measured fluorescence intensity, *f*_max_ is the maximum emission intensity at wavelength, with a full-width at half-maximal of *w* and the “skewness” is controlled by *b*. Fluorescence spectra can be accurately modeled and deconvolved by fitting to such functions. By fitting to a sum of two skewed Gaussians, we are able to accurately model the spectral component attributable to Trp emission alone (see Figure S5). We use the resulting model attributable to Trp emission, deconvolved from any contribution from iron-free heme to extract the value of CSM
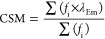
5The CSM can then be calculated more accurately. Data were collected in a thermostated cell holder (15 °C). Excitation and emission slit widths were 4 nm and C45 tryptophan emission was monitored from 325 to 550 nm. The excitation wavelength was subsequently increased in 1 nm steps for a total of 19 scans. C45 was present at a concentration of 4 μM, with some conditions containing 50% TFE (v/v). Data fitting and plotting was performed using ORIGINPRO 2019, 9.6.0.172 (MicroCal, Malvern, U.K.).

### NMR Spectroscopy

Experiments were performed on a Bruker Avance III HD 700 MHz instrument equipped with a 1.7 mm TXI Z-gradient probe at 298 K. ^15^N-labeled C45 was produced in medium supplemented with 1 g/l ^15^NH_4_Cl, as described previously.^7^ NMR spectra were recorded with 250 μM ^15^N-labeled C45 in buffer (20 mM CHES, 100 mM KCl, pH 8.6) containing 10% D_2_O. A second sample was prepared with 80% deuterated TFE, 10% buffer, and 10% D_2_O.

### EPR Spectroscopy

Low-temperature EPR spectra were recorded on a Bruker EMX (X-band) EPR spectrometer with the use of an Oxford Instruments liquid-helium system and a spherical high-quality ER 4122 (SP 9703) Bruker resonator. The instrumental conditions used to record the EPR spectra were as follows: microwave frequency ν_MW_ = 9.4677 GHz, microwave power *P*_MW_ = 0.79 mW, modulation frequency ν_m_ = 100 kHz, modulation amplitude *A*_m_ = 3 G, time constant τ = 82 ms, scan rate *V* = 0.60 G s^–1^, and number of scans per spectrum NS = 4. The slow freeze samples were prepared by immersing an EPR tube with the reacting mixture to methanol kept on dry ice. Rapid Freeze-Quenched (RFQ) EPR samples were prepared on an isopentane-free apparatus, as described previously.^49^

### Steady-State Kinetics

All reactants were preincubated in the experimental concentration of co-solvent and mixed immediately prior to measurement. Baseline measurements were subtracted from the results. Kinetics data were either collected with a plate reader (Synergy neo2 multi-mode reader by BioTek Instruments using StarLab 96-well microplates with flat-bottomed, round wells) or using a UV/vVis spectrophotometer (Agilent Cary 60 UV–Vis spectrometer) fitted with temperature regulation, in either 1 cm/3 mm/1 mm cuvettes, or mounted in a high-pressure cell (see below). All experiments for both enzymes were performed in 50 mM HEPES, pH 6.5, unless stated otherwise.

In all cases, reactions were initiated by the addition of either C45 or HRP and the formation of the ABTS radical cation was monitored over a range of wavelengths from 414 to 465 nm for which the molar extinction coefficients were calculated. The C45 temperature dependence kinetics was measured over a temperature range of 283.15–323.15 K in 5 K increments.

### Pressure Dependence Studies

An ISS high-pressure cell (ISS, Champaign, UL), fitted with a custom mounting to an absorbance spectrometer connected to a circulating water bath for temperature regulation, was used to record all pressure measurements. In all cases, reactions were initiated by the addition of HRP (0.02 nm) and the formation of the ABTS radical cation was monitored using wavelengths between 414 and 465 nm for which the molar coefficients were calculated. The experiment was carried out over a temperature range of 283.15–323.15 K in 5 K increments and a pressure range of 50–2000 bar over 9 increments.

### Pre-Steady-State Kinetics

Formation of Compound I was measured by mixing C45 with H_2_O_2_ in an Applied Photophysics SX20 Stopped-Flow spectrometer connected to a photodiode array detector and was recorded on a log-timescale. Data between 380 and 700 nm were fitted to a single exponential accounting for Compound I formation plus a linear function reflecting the decay. Data were fitted from 1 to 20 s for 80% TFE and 1 to 120 s for the data without TFE. Data were fitted globally with the rate of Compound I formation (*k*_1_) shared between all wavelengths. For TFE, a substantial background signal associated to the scattering of micelle rapture and formation during mixing was observed. The background signal was obtained by mixing C45 in 80% TFE without peroxide and subtracted from the reaction kinetics before fitting. Solvent isotope effects were determined by running the reaction in 80% D_2_O.

## Supporting Information Available

Kinetic progress curves, EPR and NMR data, raw REES data, and steady-state enzyme kinetic data (PDF)

## Abbreviations

REES: red-edge excitation shift
MMRT: macromolecular rate theory
TFE: 2,2,2-trifluoroethanol
CSM: center of spectral mass
QUBES: quantitative understanding of biomolecular edge shift
