# Supplementary material for: Rigidifying a De Novo Enzyme Increases Activity and Induces a Negative Activation Heat Capacity
Source: ACS Catal. 2021 Sep 1;11(18):11532–41. doi: 10.1021/acscatal.1c01776 (PMC8453482; doi:10.1021/acscatal.1c01776)
Supplement: Supplementary file 1 — cs1c01776_si_001.pdf [file cs1c01776_si_001.pdf]

# *Supporting Information:* Rigidifying a *De Novo* Enzyme Increases Activity and Induces a Negative Activation Heat Capacity

Sarah A Hindson,[1‡] H. Adrian Bunzel,[2,3‡] Bettina Frank,[2,4‡] Dimitri A Svistunenko,[5] Christopher Williams,[3] Marc W van der Kamp,[2] Adrian J Mulholland,[3] Christopher R Pudney,[1*] JL Ross Anderson[2*]

[1]Department of Biology and Biochemistry, Centre for Sustainable Chemical Technology, University of Bath, Bath BA2 7AY, UK. [2]School of Biochemistry, University of Bristol, Bristol BS8 1TD, UK. [3]School of Chemistry, University of Bristol, Bristol BS8 1TS, UK. [4] Bristol Centre for Functional Nanomaterials, School of Physics, University of Bristol, Bristol BS8 1TL, UK. [5]School of Life Sciences, University of Essex, Colchester CO4 3SQ, UK.

* c.r.pudney@bath.ac.uk, ross.anderson@bristol.ac.uk

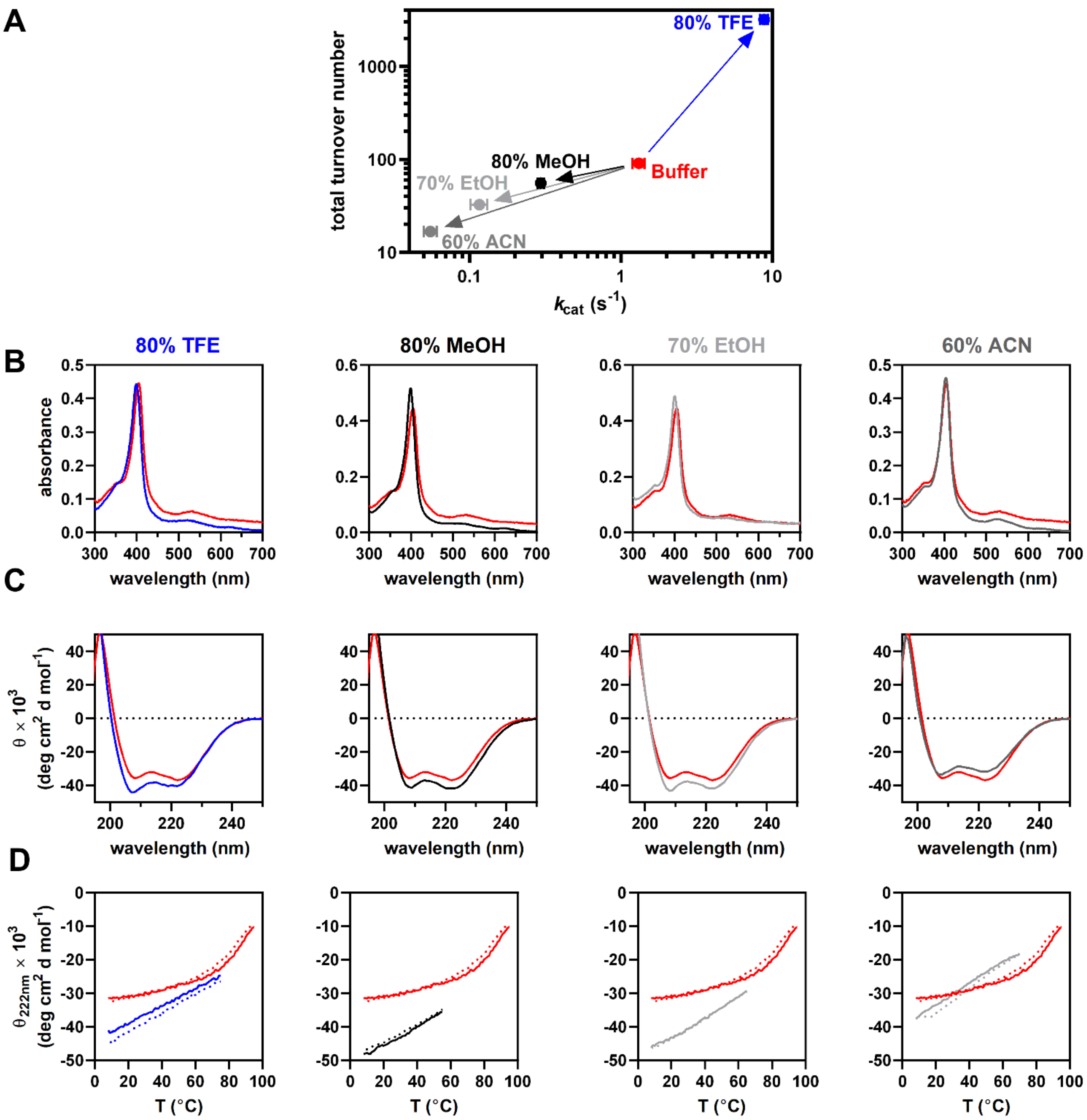


**Figure S1.** Solvent effects on C45 activity. **A,** Effect of co-solvent on TTN for C45. **B-D,** C45 absorption spectra (**B**), far-UV CD Spectra (**C**) and temperature dependence of the CD signal (**D**) in the presence and absence of different co-solvents (red; buffer, blue; TFE, black; MeOH, light grey; EtOH and dark grey; ACN).

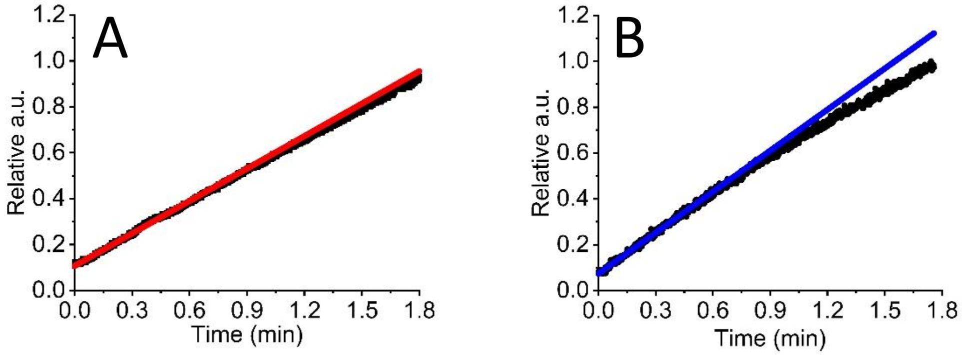


**Figure S2.** Kinetic progress curves showing the stabilising effect of TFE, $H_2O_2$ versus rate of C45 turnover in the presence (panel **A**) and absence of TFE (panel **B**). Solid lines are the fit of a simple linear function to the firs 0.5 min of data, showing the deviation from linearity (initial rate) with respect to time. *Conditions,* 0.15 $\mu$M C45 and 73 mM ABTS in 50 mM HEPES buffer, pH 6.5 at 30 °C.

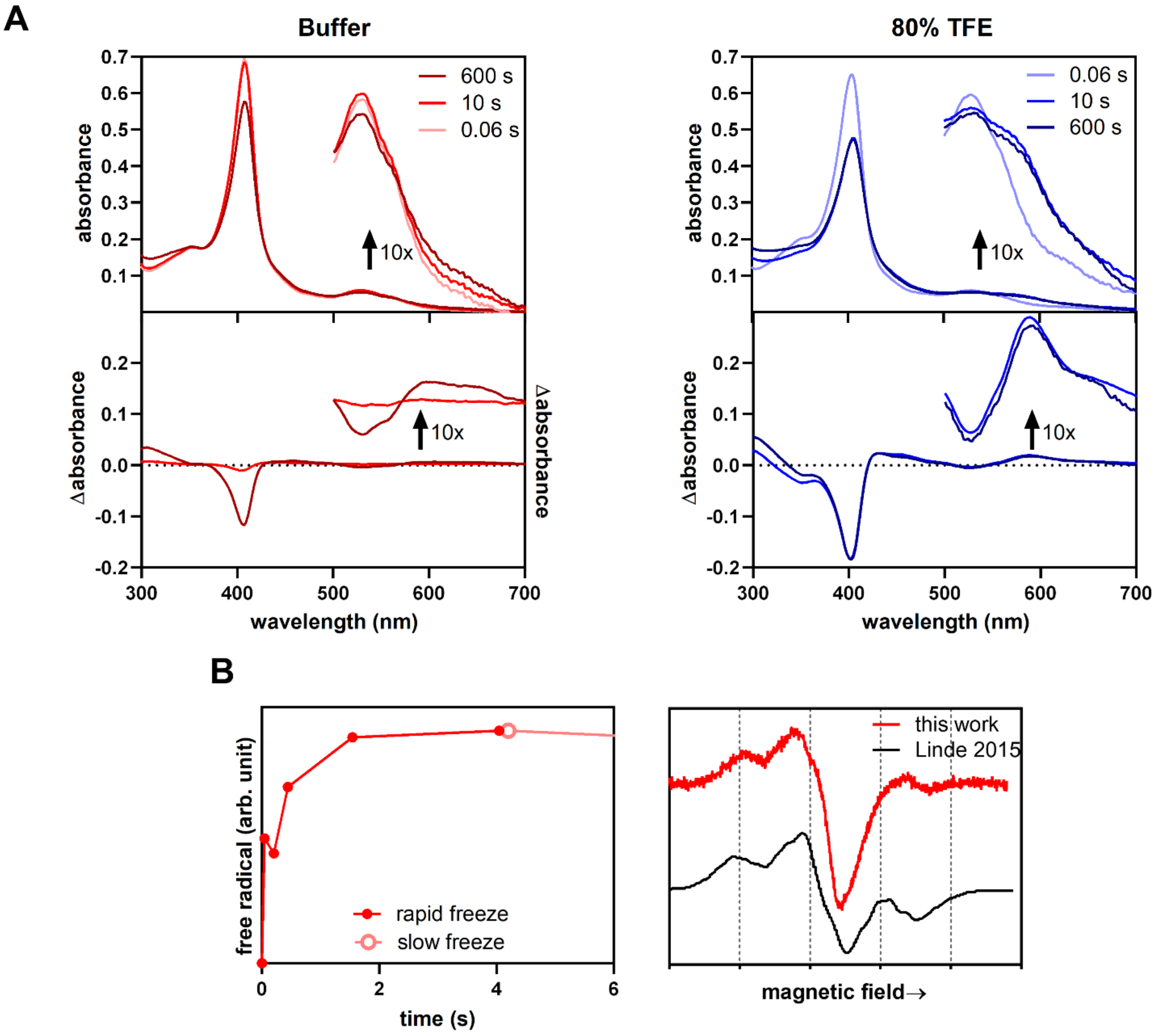


**Figure S3.** Monitoring Compound I formation. **(A)** Rapid mixing of peroxide and C45 in buffer (left, red) and 80% TFE (right, blue) leads to formation of Compound I, as indicated by the UV/Vis spectra (top). To illustrate spectral changes associated with the reaction, difference spectra compared to the first time point at 0.06 s are shown (bottom). More of the intermediate-associated Q-band at 586 nm is present after 10 s in TFE compared to buffer, signalling rapid formation of Compound I in the presence of the cosolvent. The Q-band and Soret peak degrade much slower in TFE compared to buffer, indicating that Compound I is more stable in the fluorinated solvent. **(B)** Detailed view of the initial part of the kinetic curve of the radicals presented in Figure 2E. The slow and rapidly freeze-quenched samples have different densities (and therefore different EPR signals intensities); the two sets of data have been brought to a common value at the common time point – 4 s. The unusual, three component EPR spectrum of C45 (red) is plotted on a common magnetic field scale with the published spectrum of a dye decolorizing peroxidase (DyP) assigned to a Trp radical.[22] The spectrum of DyP from ref 22 (black) has been digitized using UN-SCAN-IT 6.0 (Silk Scientific). As the microwave frequencies used in ref 22 and in this study are slightly different, the EPR signals appear at slightly different values of the magnetic field (horizontal axis). Because of that, the values of the field, increasing from left to right, are not shown in the figure, but rather the vertical gridlines at a 20 Gauss intervals indicate the overall scale of the scan. The two spectra have been aligned by a common value of the g-factor.

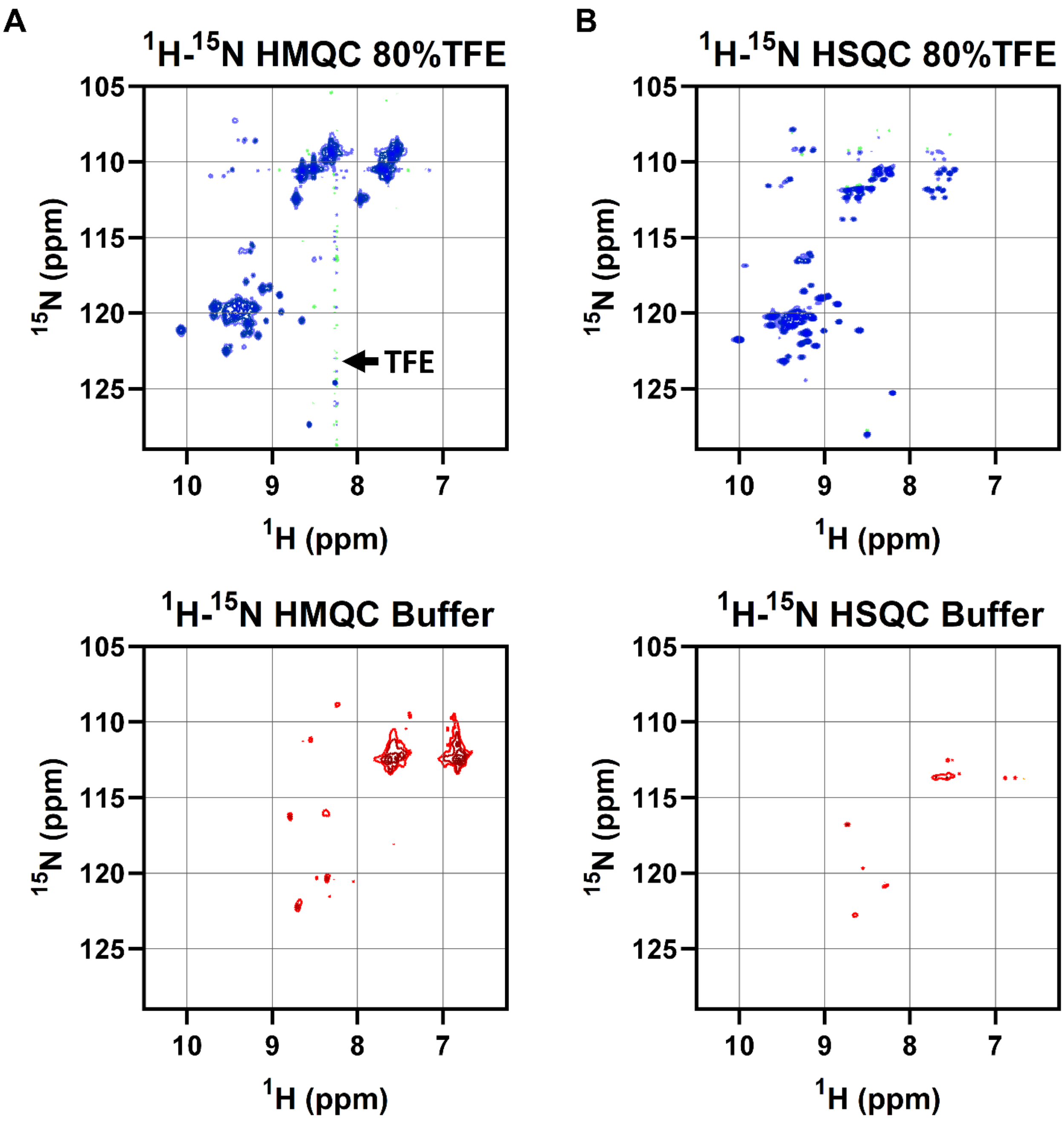


**Figure S4**. NMR spectra of C45 in 80% TFE (top, blue) and buffer (bottom, red) **A)** $^{1}$H-$^{15}$N HMQC (left) and **B)** HSQC (right) spectra become more disperse and more peaks are apparent after addition of 80% TFE.

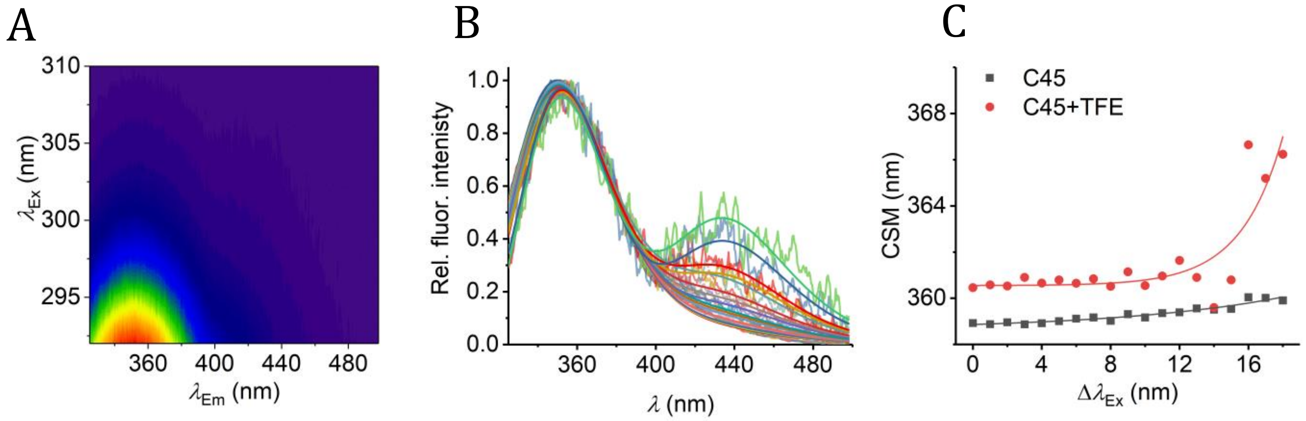


**Figure S5**. REES spectroscopy and extracting QUBES data. **A,** Raw REES data. **B,** Fitting of raw REES data in panel A using Eq4 as described in *Methods.* **C,** Plot of CSM *versus* excitation energy for C45 in the presence and absence of 50% TFE. Solid lines show fits to Eq 2. *Conditions,* 4 $\mu$M C45 in 50 mM HEPES buffer, pH 6.5, 15°C.

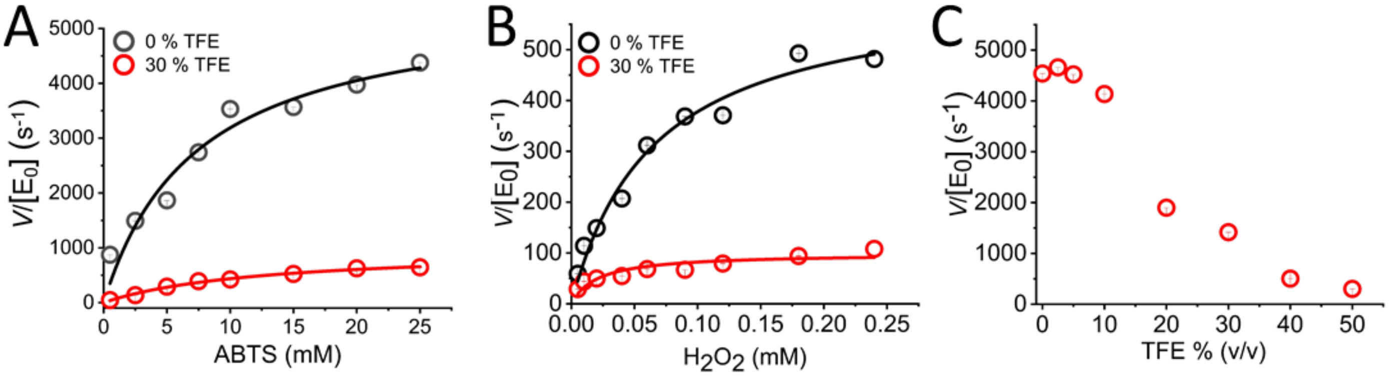


**Figure S6.** The steady state kinetics of HRP turnover at 0% and 30% TFE recorded over a range of ABTS and $H_2O_2$ concentrations (panel **A** and **B** respectively). **C**, Effect of increasing TFE % (v/v) on maximal rate of HRP, all data was recorded in triplicate and SE is shown. *Conditions,* Panel A 0.5 nM HRP, 0.5 mM $H_2O_2$, Panel B 0.2 nM HRP, 30 mM ABTS and panel C 0.2 nM HRP, 0.5 mM $H_2O_2$ and 30 mM ABTS, all in 50 mM HEPES buffer, pH 6.5.

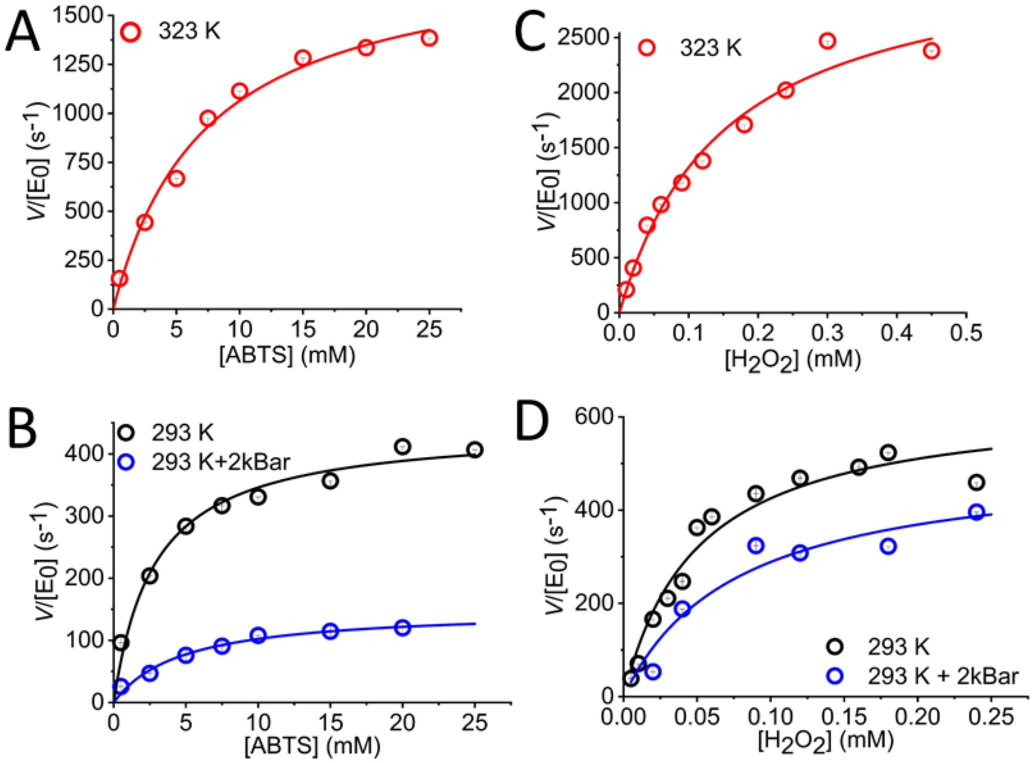


**Figure S7.** The steady state kinetics of HRP turnover varying [ABTS] (panel **A and B**) and [$H_2O_2$] (panel **C and D**), All data were recorded in triplicate and error bars represent the standard error. *Conditions,* 0.5 nM HRP, 0.13 mM $H_2O_2$ (panel A), 30 mM ABTS (panel B) in 50 mM HEPES buffer, pH 6.5.

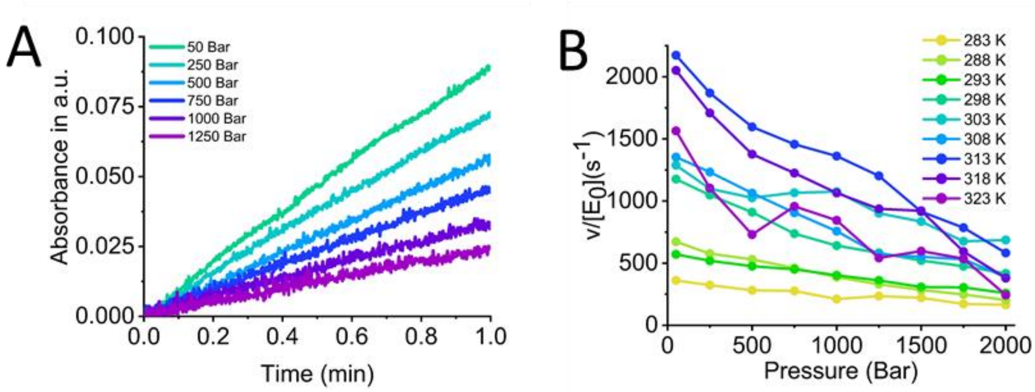


**Figure S8.** Combined pressure and temperature dependence of HRP turnover. **A,** Raw data shows HRP progress curves are linear at elevated pressures. **B,** Plot of $v/[E_0](s^{-1})$ over entire pressure and temperature range of HRP, data was recorded in triplicate. *Conditions,* 0.2 *n*M HRP, 1.8 mM $H_2O_2$ and 73 mM ABTS in 50 mM HEPES buffer, pH 6.5.
